# Chronic Consumption of a Commercial Energy Drink Reduces Blood Pressure in Normotensive Wild-Type Mice

**DOI:** 10.3389/fnut.2019.00111

**Published:** 2019-07-23

**Authors:** Liam Graneri, Zachary D'Alonzo, Virginie Lam, John Mamo, Satvinder Dhaliwal, Ryusuke Takechi

**Affiliations:** ^1^Curtin Health Innovation Research Institute, Curtin University, Perth, WA, Australia; ^2^Faculty of Health Sciences, School of Pharmacy and Biomedical Sciences, Curtin University, Perth, WA, Australia; ^3^Faculty of Health Sciences, School of Public Health, Curtin University, Perth, WA, Australia

**Keywords:** blood pressure, caffeine, coke, energy drink, sugar free energy drink, taurine, vitamin B

## Abstract

**Objective:** Studies report that acute consumption of energy drinks transiently increases blood pressure (BP). However, few studies report the effect of chronic energy drink consumption on BP. In this study, we investigated the effects of long-term energy drink ingestion on BP in C57BL/6J normotensive wild-type mice.

**Research Methods and Procedures:** Groups of mice were randomized to no treatment (water) (Control group), or to Mother^™^ provided as a decarbonated 30% (v/v) drinking solution (Energy Drink group), sugar-free Mother^™^ at 30% (Sugar-free group), Coca Cola^™^ at 30% (Coke group) for a total intervention period of 13 weeks.

**Results:** After 13 weeks of intervention, the control mice showed a modest increase in systolic blood pressure (SBP), diastolic blood pressure (DBP), and mean arterial pressure (MAP) by 7.1 ± 8.8, 5.8 ± 9.4, and 6.3 ± 9.1 mmHg, respectively. However, the Energy Drink significantly decreased the DBP and MAP by 18.8 ± 9.9 and 15.3 ± 9.8 mmHg, respectively. Similarly, Sugar-free group mice showed significant decrease of the SBP, DBP, and MAP by 10.85 ± 5.6, 18.7 ± 6.7, and 15.6 ± 6.1 mmHg, respectively. The SBP, DBP, and MAP in Coke mice showed no significant changes. The estimated cumulative intake of caffeine, taurine, and vitamin B3 and B5 was significantly higher in the mice of Energy Drink and Sugar-free groups compared to the Control and Coke mice.

**Conclusion:** Collectively, the data suggest that the long-term chronic consumption of energy drinks may significantly lower the BP in normotensive mice through the actions of caffeine, taurine, and/or B-vitamins. The study findings do not support consideration of energy drinks for BP management, but rather demonstrate no long-term amplification of BP in normotensive preclinical models.

## Introduction

Popularity of caffeinated energy drinks is markedly increasing, particularly amongst adolescents, and young adults. Indeed, a recent survey study for university students in the US revealed that 51% of students regularly consume caffeinated energy drinks, particularly during the semester teaching period (1). A growing number of recent clinical randomized controlled trials and animal intervention studies report some profound detrimental effects of energy drinks on cardiometabolic conditions. Acute consumption of energy drink (~1,000 ml in 45 min) by young healthy individuals induced a significant prolongation of QTc interval and an elevation of systolic blood pressure (SBP) within 2 h of the beverage intake (2). Additionally, a study involving 24 patients with familial long QT syndrome showed a significant increase in QTc interval and an acute increase of systolic and diastolic blood pressure (DBP) following a singular challenge with consumption of an energy drink. The latter study also noted that of 24 subjects participated, 3 individuals had >50 ms QTc prolongation following the energy drink intake, imposing a potentially fatal event (3). In a study with adult wild-type Wistar rats, an acute single oral gavaging of energy drink in combination with alcohol induced renal nephrotoxicity and hepatic hydropic and hyaline degenerations (4). However, the majority of previous studies have only tested short- to mid-term cardiovascular effects of acute energy drink consumption, and no studies to date have investigated long-term physiological effects. Therefore, in the present study, we explored for the first time the putative effects of chronic energy drink consumption on blood-pressure in normotensive wild-type mice.

## Methods

### Animal Intervention

All animal protocols were approved by Institutional Animal Research Ethics Committee (approval no. 2018–3). Male 6-week old C57BL/6J mice were purchased from Animal Resources Center (WA, Australia), and allocated to Control group receiving water, Energy Drink group receiving 30% (v/v in water) Mother^™^, Sugar-Free group receiving sugar-free Mother^™^ 30% (v/v), or Coke group receiving Coca Cola^™^ 30% (v/v) for 13 weeks. Coke group was added in comparison to Mother^™^ as a widely consumed, lightly caffeinated sugar drink that is produced by the same company as Mother^TM^ and we considered that this would be of high research interest and significance. Mice in all groups were maintained on standard AIN93M maintenance chow (Specialty Feeds, WA, Australia) and had 24 h *ad libitum* access to chow and drinks. Major nutritional composition of chow, Mother^™^, sugar-free Mother^™^, and Coca Cola^™^ is presented in Table 1. The consumption of energy drink and chow was monitored twice a week.

**Table 1:** Nutrition composition table of energy drinks and chow.

**Per 100 g or 100 ml**	**mother™**	**Sugar-free Mother™**	**Coca cola™**	**Chow (AIN93M)**
Energy (kJ)	191	19	180	1570
Carbohydrate (mg)	10.1	0.1	10.6	64.8
Fat (mg)	0	0	0	4
Caffeine (mg)	31.9	31.8	9.7	0
Taurine (mg)	400	400	0	0
Vitamin B3 (mg)	1.8	1.8	0	3
Vitamin B6 (mg)	0.2	0.2	0	0.7
Vitamin B12 (μg)	0.5	0	0	10.3
Vitamin B5 (mg)	0.66	0.66	0	1.65

### Blood Pressure Measurement

At baseline (week 0) and after 13 weeks of the energy drink intervention, SBP, DBP, and mean arterial pressure (MAP) were measured by using CODA tail-cuff rodent blood pressure system as described previously (5, 6). Briefly, each mouse was gently restrained in acrylic restrainer, tail cuffs were placed on the tail, and the mice were left on an infrared heater for 5 min until the tail temperature was raised to ~37°C. After five acclimatization measurements, ten blood pressure measurements were taken with 5 s intervals. The mean of ten measurements was calculated per mouse.

### Statistical Analysis

In order to produce sufficient statistical power to detect the effects of energy drinks on blood pressure, 10 mice per group were included in the study based on previous studies (6). Cumulative intake of chow, energy drink, and its nutrients over the experimental period of 13 weeks were estimated with area under curve (AUC). All data was expressed as mean ± SD and was normally distributed, and statistical significance was determined by two-tailed paired *t*-test, one-way ANOVA with Tukey's *post-hoc* test, or two-way ANOVA at *p* < 0.05.

## Results

The energy drink interventions were well-tolerated and there were no adverse events. Average weight gain of mice received Control chow for 13 weeks was 9.2 ± 0.5 g (Supplementary Figure S1). The mice in Energy Drink group gained 8.0 ± 0.6 g of weight, whilst Sugar-free group gained 9.7 ± 0.7 g. The mice of Coke group gained 9.3 ± 0.6 g of weight. There were no significant differences in weight gain across all groups.

**Figure 1:**
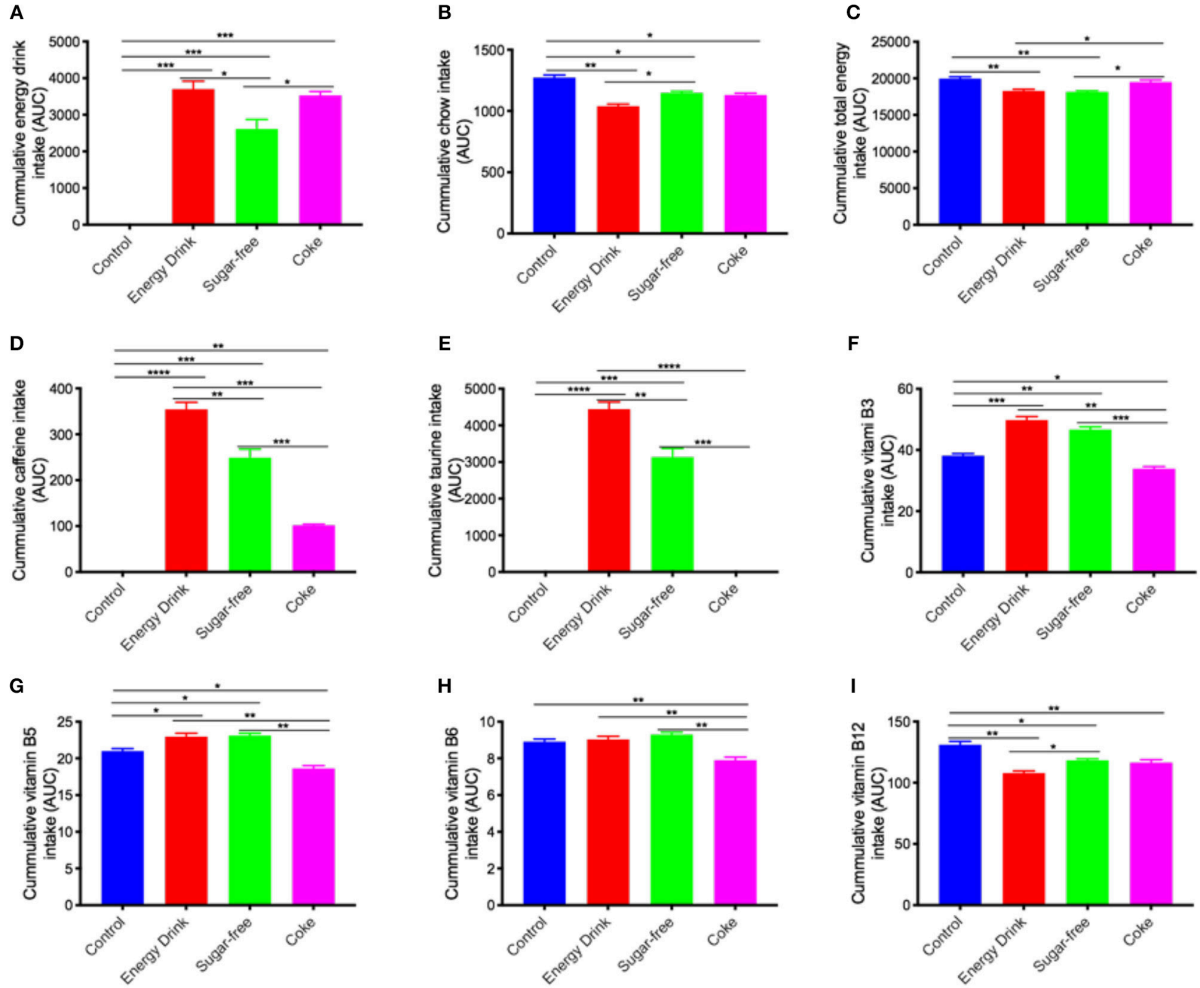
Cumulative energy drink and chow consumption and estimated dietary intake. The mice received normal water (Control), Mother^™^ (Energy Drink), sugar-free Mother^™^ (Sugar-free), or Coca Cola^™^ (Coke) for 13 weeks. The cumulative intake of drink, chow, and nutrients over the entire 13-week experimental period is shown as area under curve (AUC) (also see Supplementary Figure S2). **(A,B)** The consumption of energy drinks and chow were measured weekly. Based on the drink and chow consumption, the intake of total energy **(C)**, caffeine **(D)**, taurine **(E)**, vitamin B3 **(F)**, vitamin B5 **(G)**, vitamin B6 **(H)**, and vitamin B12 **(I)** was estimated per kg weight. The statistical significance was assessed by one-way ANOVA with Tukey's *post-hoc* test (*n* = 10, *p < 0.05, **p < 0.01, ***p < 0.001, ****p < 0.0001).

Over the 13-week intervention period, mice maintained on full complement Mother^™^, or full complement Coca Cola^™^ drank ~20% more beverage than mice maintained on sugar-free Mother^™^. Sugar-free group mice (Figure 1A). There was a compensatory decrease in the amount of chow consumed with greatest decrease observed in Energy Drink > Coke > Sugar-free, respectively (Figure 1B). Net cumulative energy intake (drink + chow) is presented in Figure 1C and was significantly lower in the Energy Drink group compared to Control or Coke group.

The Control mice consumed no caffeine (Figure 1D). The cumulative caffeine intake of Mother^™^ was significantly greater than the sugar-free Mother^™^ because of greater consumption and more than 2-fold greater than the Coke group of mice. The cumulative caffeine intake of Sugar-free group was also substantially greater than the Coke treatment group. Similarly, the cumulative intake of taurine was greatest in Energy Drink > Sugar-free > Coke or Control mice, the latter two groups with no exogenous provision through diet (Figure 1E).

Vitamin B is indicated in rodent chow formulations, nonetheless, provision of either Energy Drink or Sugar-free drinking solutions exaggerated total vitamin B3 and vitamin B5 intake, despite a reduction in chow consumption (Figures 1F,G). The cumulative intake of vitamin B6 in the Energy Drink groups of mice was comparable to chow and water fed Controls, but significantly reduced in Coke mice (Figure 1H). The cumulative intake of vitamin B12 in Energy Drink and Sugar-free, and Coke group was reduced by ~10% compared to water fed Controls (Figure 1I).

**Figure 2:**
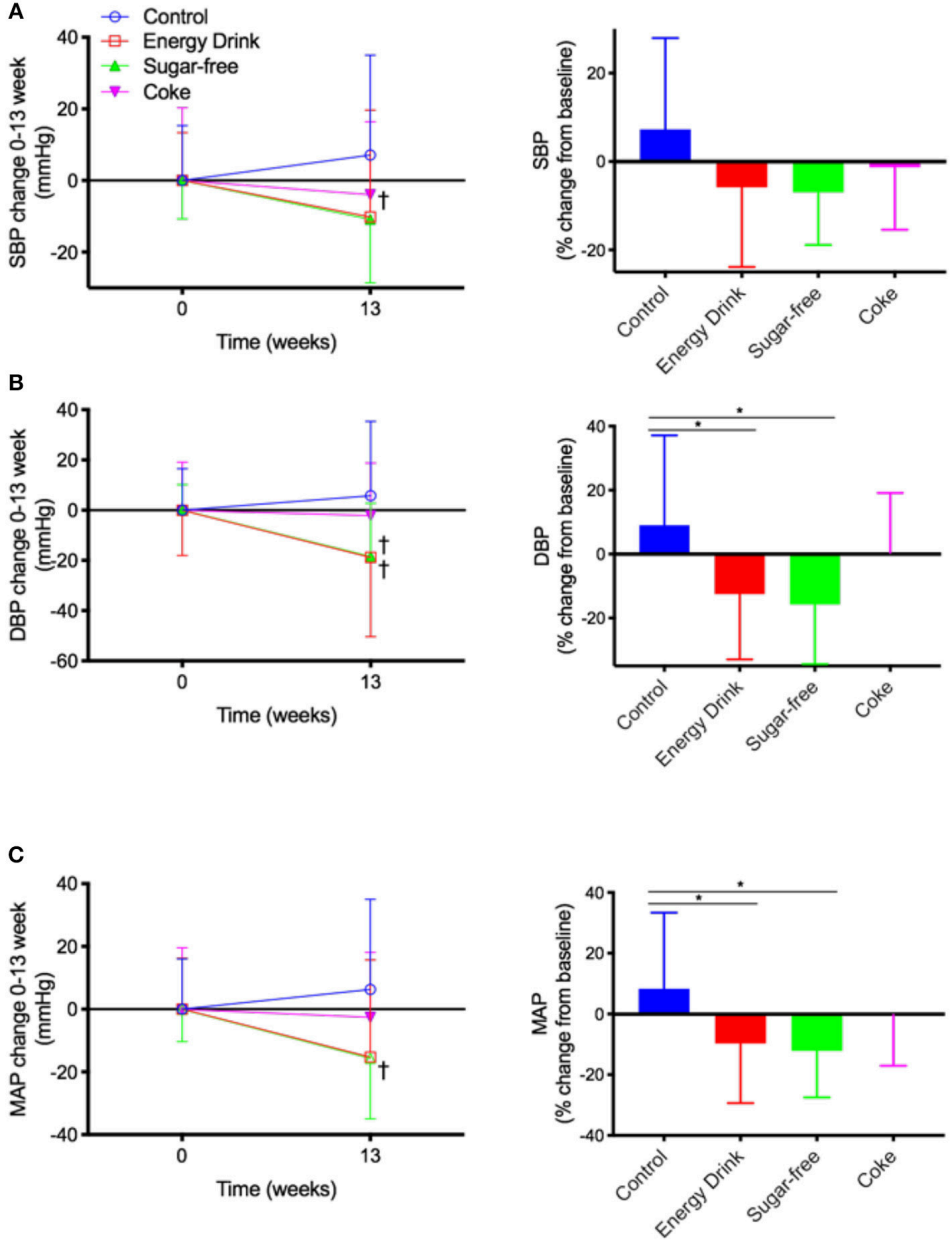
Blood pressure. The mice received normal water (Control), Mother^™^ (Energy Drink), sugar-free Mother^™^ (Sugar-free), or Coca Cola^™^ (Coke) for 13 weeks. **(A)** Systolic blood pressure (SBP), **(B)** diastolic blood pressure (DBP), and **(C)** mean arterial pressure (MAP) were measured with tail-cuff method. Raw measurements of SBP as well as percent change from 0 to 13 weeks are presented. The statistical significance was assessed by one-way ANOVA with Tukey's *post-hoc* test (*n* = 10, *p < 0.05), or paired two-tailed *t*-test (*n* = 10, ^†^*p* < 0.05).

After 13 weeks of intervention, Control mice received only water had a modest increase in SBP by 7.1 ± 8.8 mmHg (+7.3% increase) compared to their baseline measurement (Figure 2A). In contrast, the SBP of mice maintained on Energy Drink for 13 weeks showed non-significant decline in SBP of 10.2 ± 11.6 mmHg (−5.9%, *p* = 0.09). The SBP of Sugar-free mice was significantly reduced by 10.85 ± 5.6 mmHg (−7.0%). The mice in Coke group showed no significant change in SBP.

The DBP of Control mice was modestly increased by 5.8 ± 9.4 mmHg (+9.0%) compared to their baseline (Figure 2B). The mice maintained on full complement Mother^™^ had a significant decrease in DBP by 18.8 ± 9.9 mmHg (−12.5%). Similarly, the DBP of mice maintained on sugar-free Mother^™^ showed significant decrease by 18.7 ± 6.7 mmHg (−15.7%). The DBP of Coke group mice did not significantly alter from their baseline.

The MAP of control mice increased by 6.3 ± 9.1 mmHg (+8.3%) over 13 weeks of Figure 2C. The MAP of the mice receiving Energy Drink was reduced significantly by 15.3 ± 9.8 mmHg (−9.7%), and the change rate was significantly different from the Control group. The Sugar-free treatment group mice also had a significant reduction in MAP by15.6 ± 6.1 mmHg (−12.1%). The MAP in Coke group was comparable to its baseline.

## Discussion

A number of studies show that acute ingestion of energy drinks significantly increases blood pressure, however no studies to date considered longer-term effects of chronic consumption in normotensive individuals, or preclinical models. The chronic ingestion of energy drinks is an important public health consideration given the markedly increased consumption of highly caffeinated beverages particularly amongst adolescents and young adults. In this study, the putative effects of long-term consumption of a widely available energy drink Mother^™^ on BP was studied in normotensive wild-type mice.

The Control group mice maintained on chow and water had a modest increase in SBP, DBP, and MAP. This observation is consistent with previous reports showing an age-associated increase in BP due to oxidative stress-mediated changes in renin-angiotensin system and increase in vascular smooth muscle tone (7, 8).

In this study, chronic 13-week consumption of Mother^™^ energy drink formulations with/without sugar demonstrated substantial reductions in SBP, DBP, and MAP. The equipotency of full complement Mother^™^ and sugar-free Mother^™^ with generally decreased consumption of ordinary chow, suggests that ingredients other than sugar found in Mother^™^ formulations are associated with longer-term attenuation of SBP, DBP, and MAP in normotensive mice. Consistent with the results indicated, Shah et al. reported that in healthy volunteers, there was no significant changes in BP when subject consumed energy drinks twice daily for 7 days, despite an acute increase in systolic and diastolic BP within 1–5 h of the post ingestion period (9). However, the intervention period was substantially shorter in the latter study. In this study, BP was determined at 11 a.m. on test days and not fasted, ~5 h after the ordinary nocturnal feeding cycle. The data presented therefore represents acute and adaptation responses to longer-term feeding, which is physiologically relevant. Furthermore, the Mother group showed decrease in caffeine intake during the final week of the study, which may also be responsible for the reduction of BP at 13 weeks. The study is the first to demonstrate substantial hypotensive effects of long-term energy drink intake in mice that were otherwise healthy and normotensive at commencement of intervention.

In contrast to the both Energy Drink groups, no changes in SBP, DBP, or MAP were observed in the mice that were maintained on standard Coke for 13 weeks. This contradicts with previous findings in wild-type rats, where significant SBP elevation was observed with chronic coke ingestion (10–12). Such conflicting findings may be explained by the species difference. Indeed, rats are more prone to sugar-enriched dietary interventions (13). Furthermore, the previous rat studies using coke used undiluted coke whilst the current study used 30% diluted coke for more clinical relevance. Moreover, the BP measurement method employed by these studies was cuffless plethysmography, which is substantially different from the present study. Due to these substantial differences in the study conditions, the data may not be directly comparable.

Caffeine is one of the potential agents found in commercial energy drinks that may be responsible for altering BP. Caffeine is reported to increase blood pressure acutely primarily by enhancing sympathetic nerve activity and raising heart rate. A clinical randomized controlled study showed that a single oral caffeine intake at 3.3 mg/kg dose significantly raised SBP and DBP in both healthy participants and hypertensive patients (14). However, a population study reported that habitual caffeine intake was not significantly associated with the BP changes in adolescents (15). Long-term effects of caffeine were studied in an OLETF rat model of diabetes, reporting that caffeine treatment for 4 weeks significantly decreased systolic and DBP and indeed improved diabetic nephropathy (16). However, the caffeine dosage used in this study was 90.7 mg/kg/day, which is markedly higher than the current study. Yu et al. demonstrated that long-term 15-day caffeine intake reduced blood pressure by attenuating renal epithelial Na+ channel expression and activity and promoting urinary sodium excretion (17). Consistent with these observations, in the present study, the mice in Energy Drink and Sugar-free groups showed marked reductions in BP measures. Alternatively, other components of the Mother^™^ formulation may have been responsible or longer-term BP lowering effects. Consistent with the latter, sugar free Mother^™^ was equipotent to full complement Mother^™^, despite significantly less caffeine being consumed over the intervention group. Similarly, Coke treated mice, with approximately one half of caffeine intake compared to Sugar-free showed no reduction in BP measures. Threshold effects of caffeine cannot be determined from these study findings.

Taurine is another potential hypotensive component of commercial energy drink, with substantial quantities indicated in most commercial drink formulation. Relevant to this study, Mother^™^ formulations contain a remarkable 2,000 mg per 500 mL unit. Taurine is a ubiquitous amino-sulfonic acid found in abundance in animal tissue and has been reported to exert multiple cardioprotective effects including improvement of cardiac function, reduction of plasma LDL, cholesterol and triglycerides, prevention of overweight, and attenuation of blood pressure (18). A clinical randomized controlled study revealed that 6 g/day of taurine intake significantly lowers the blood pressure within 7 days in hypertensive subjects (19). Taurine's hypotensive effects has also been demonstrated in preclinical models of hypertension including spontaneously hypertensive rats (SHR), salt-sensitive Dahl rats and fructose-induced hypertensive rats (20). The preclinical studies used taurine dosage at 1–5% in drinking solution, which is >10-fold greater than the current study where the estimated taurine concentration was 0.12% for the Energy Drink and Sugar-free groups. Interestingly, Trachtman et al. reported that in SHR rats, the arterial pressure was significantly reduced after 4 weeks of taurine treatment, but its anti-hypertensive effects reached threshold at 16 weeks an adaptive response with longer duration (21). In accordance with these observations, the mice provided Mother^™^ formulations in the present study with substantial ingestion of taurine showed significant BP reduction.

Some B-vitamins are also reported to alter BP. A clinical study reports that in healthy individuals, nicotinamide (vitamin B3) reduces BP in long-term (22). A cross sectional study of male adolescents revealed that the plasma concentration of vitamin B12 is inversely associated with SBP, whilst plasma vitamin B6 is positively associated with SBP (23). Vitamin B12 deficiency often observed in vegetarians are reported to be associated with elevated BP (24). The intake of vitamin B12 is also reported to be associated with lower blood pressure in preschool children (25). The deficiency of vitamin B5 is shown to correlate with hypertension (26). In contrast, in a rat model of vitamin B6 deficiency, no changes of BP were observed (27). In the current study, Energy Drink and Sugar-free treated groups had significantly higher cumulative intake of vitamin B3 and B5, whereas the B12 intake was higher in Control mice. The vitamin B6 intakes were comparable across all the groups. These data suggest that in normotensive wild-type mice, vitamin B3 and B5 may have significant effects in reducing the BP.

However, the study did not measure blood biomarkers or vascular pathological changes and thus, is unable to determine the exact mechanistic pathways responsible for the hypotensive effects of chronic energy drink consumption. Future studies are necessary to confirm such potential underlying mechanisms.

## Conclusions

Our findings collectively suggest that if sympathetic modulation of vascular tone adapts to regular ingestion of highly caffeinated energy drink beverages, the combined provision of caffeine/taurine/B-vitamins independently, or synergistically lowers blood pressure in normotensive otherwise healthy wild-type mice. Potential effects of reduced blood pressure in chronic consumers of energy-drink formulations may be clinically relevant.

## Data Availability

All datasets generated for this study are included in the manuscript and/or the Supplementary Files.

## Ethics Statement

All animal protocols were approved by Institutional Animal Research Ethics Committee, Curtin University (approval no. 2018–3).

## Author Contributions

The study was designed by VL, JM, SD, and RT. Animal experiments and sample collections were done by LG, ZD'A, VL, and RT. Data analyses and manuscript writing was done by LG, ZD'A, VL, JM, SD, and RT.

### Conflict of Interest Statement

The authors declare that the research was conducted in the absence of any commercial or financial relationships that could be construed as a potential conflict of interest.

## Supplementary Material

Supplementary Figure S1 Weight and weight gain.

Supplementary Figure S2 Average energy drink and chow consumption and estimated dietary intake.
