# Supplementary figures and images for: Chronic Consumption of a Commercial Energy Drink Reduces Blood Pressure in Normotensive Wild-Type Mice
Source: Front Nutr. 2019 Jul 23;6:111. doi: 10.3389/fnut.2019.00111 (PMC6663975; doi:10.3389/fnut.2019.00111)

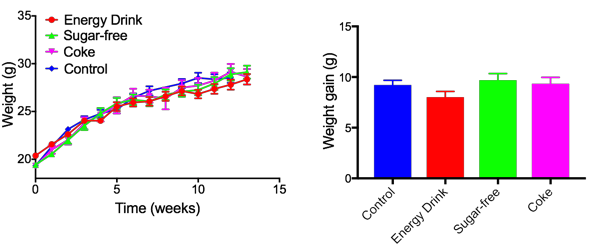

Supplement: Supplementary Figure S1 — Weight and weight gain. [file Image_1.tiff]

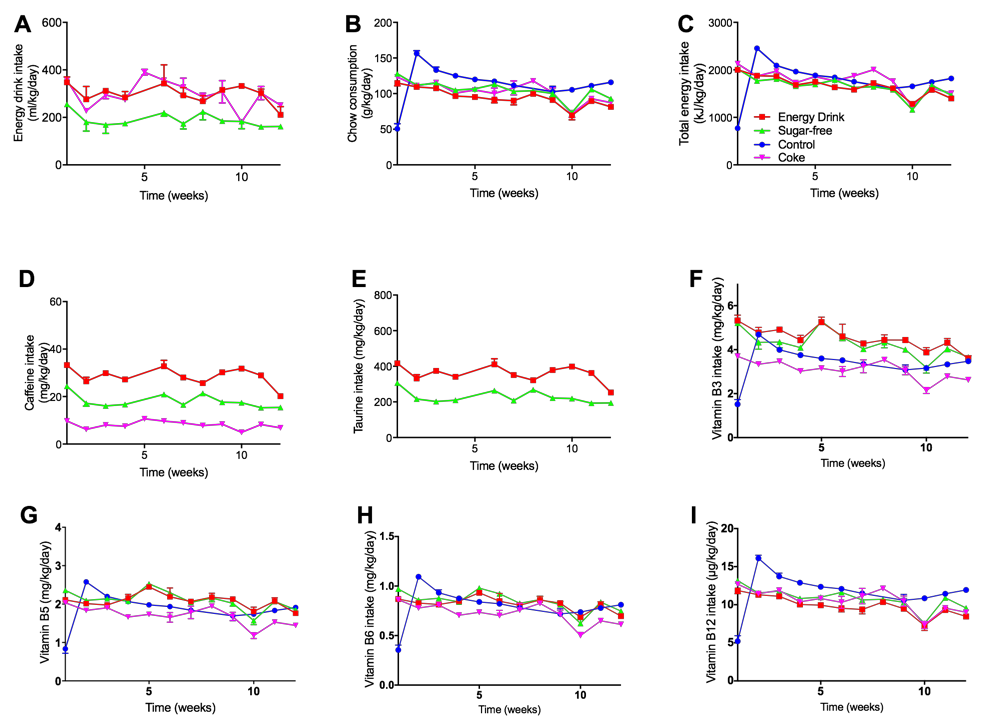

Supplement: Supplementary Figure S2 — Average energy drink and chow consumption and estimated dietary intake. [file Image_2.tiff]
